# Comparative Study of Experimental and Modeling of Fly Ash-Based Concrete

**DOI:** 10.3390/ma15113762

**Published:** 2022-05-24

**Authors:** Kaffayatullah Khan, Ayaz Ahmad, Muhammad Nasir Amin, Waqas Ahmad, Sohaib Nazar, Abdullah Mohammad Abu Arab

**Affiliations:** 1 Department of Civil and Environmental Engineering, College of Engineering, King Faisal University, Al-Ahsa 31982, Saudi Arabia; mgadir@kfu.edu.sa (M.N.A.); 219041496@student.kfu.edu.sa (A.M.A.A.); 2 MaREI Centre, Ryan Institute and School of Engineering, College of Science and Engineering, National University of Ireland, H91 TK33 Galway, Ireland; a.ahmad8@nuigalway.ie; 3 Department of Civil Engineering, COMSATS University Islamabad, Abbottabad 22060, Pakistan; waqasahmad@cuiatd.edu.pk (W.A.); sohaibnazar@cuiatd.edu.pk (S.N.)

**Keywords:** concrete, fly ash, modeling, machine learning, compressive strength

## Abstract

The application of supplementary cementitious materials (SCMs) in concrete has been reported as the sustainable approach toward the appropriate development. This research aims to compare the result of compressive strength (C-S) obtained from the experimental method and results estimated by employing the various modeling techniques for the fly-ash-based concrete. Although this study covers two aspects, an experimental approach and modeling techniques for predictions, the emphasis of this research is on the application of modeling methods. The physical and chemical properties of the cement and fly ash, water absorption and specific gravity of the aggregate used, surface area of the cement, and gradation of the aggregate were analyzed in the laboratory. The four predictive machine learning (PML) algorithms, such as decision tree (DT), multi-linear perceptron (MLP), random forest (RF), and bagging regressor (BR), were investigated to anticipate the C-S of concrete. Results reveal that the RF model was observed more exact in investigating the C-S of concrete containing fly ash (FA), as opposed to other employed PML techniques. The high R2 value (0.96) for the RF model indicates the high precision level for forecasting the required output as compared to DT, MLP, and BR model R^2^ results equal 0.88, 0.90, and 0.93, respectively. The statistical results and cross-validation (C-V) method also confirm the high predictive accuracy of the RF model. The highest contribution level of the cement towards the prediction was also reported in the sensitivity analysis and showed a 31.24% contribution. These PML methods can be effectively employed to anticipate the mechanical properties of concretes.

## 1. Introduction

CO_2_ emissions from industry, transportation, and services, and nitrogen and methane oxides from agriculture are significant greenhouse gases (GHGs) [1]. Worldwide worries about the environmental, economic, and social consequences of GHG emissions such as CO_2_ have prompted the growth and deployment of a variety of CO_2_ emission mitigation technologies and initiatives [2,3,4,5,6,7]. At this time, environmental sustainability has developed as a global objective for social interests [8,9,10]. Furthermore, ecological issues about CO_2_ ejection from the Ordinary Portland Cement (OPC) manufacturing process have prompted past academics to look at the viability of other materials to substitute OPC during concrete production [11,12,13]. According to a study [14], the use of waste materials is desirable for the sustainability of the construction sector; however, another study [15] claims that the application of byproducts obtained from industries as a supplementary cementitious material (SCM) partly substitute OPC has substantially helped to achieve a more green environment. The growing demand for the strength properties along with the durability of concrete has necessitated the incorporation of a variety of industrial wastes with pozzolanic attributes into the OPC [16,17,18,19,20,21]. Additionally, these components used in OPC have a remarkable result in the microstructure alteration of cement pastes and the physio-mechanical parameters of concretes [19,22,23]. The application of waste products in concrete structures not only decreases ecological pollution but also improves the fresh and hardened properties of the selected concrete [22,23,24,25,26,27]. Due to these aspects, waste materials are frequently employed to improve the characteristics of concrete [28,29]. Nowadays, industrial wastes of various sorts and nanoparticles are employed in concrete [30,31]. A set of the waste materials frequently incorporated in concrete from the industries are ground granulated blast furnace slag, metakaolin, fly ash, and silica fume. However, nano industrial wastes which are frequently using in concrete are graphene, nano silica, titania–silica nanosphere, nano titanium, carbon nanotubes, and nano metakaolin.

FA is one of the most utilized SCM in concretes [32,33,34,35,36]. The FA obtained from coal incineration activities is not risky from the radiological fact [37]. Regrettably, it comprises trace levels of hazardous substances derived from coal-burning, including mercury, fluorine, and [38]. After burning, approximately 10–40% of chlorine and fluorine and 30–80% of mercury in coal are reported to retain in FA [39,40]. As a result, this industrial waste can be classified as a possibly hazardous substance in some instances. FA is an effective, very desirable waste for recycling purposes since concretes containing these supplements in proportions of up to 20% as OPC substitutes exhibit enhanced stability and fracture toughness [41,42,43,44], deterioration resistance [45], and tolerance to elevated temperatures [46]. Additionally, by utilizing FA, eco-friendly green material for civil engineering might be produced [47,48,49,50,51] and promote the development of a specific microstructure in concrete matrices, thereby facilitating the restriction of harmful elements [52]. Initially, the usage of FA in concrete enables the reduction of problematic disposal sites associated with this waste. It is worth noting that about 800 million tons of FA are generated annually on a global scale [53,54]. Due to the huge volume of combustion byproducts and their lack of usage, the necessity for dry or wet landfill sites to be constructed, maintained, and secured arises. It is a considerable environmental and public issue since the resulting contamination of the atmosphere has a detrimental effect on people’s health and well-being and might contribute to the development of severe environmental infections. Dumping huge amounts of FA in landfilling is also detrimental, as they are extremely light and fine in dry conditions, making them easily dispersed by wind. Thus, the substitution of FA cement is an unambiguously environmentally acceptable alternative.

Moreover, it is necessary to introduce soft computing methods to accurately forecast the nature and performance/strength of materials. Artificial intelligence (AI) approaches are gaining more popularity in this aspect which are usually introduced to estimate the various characteristics of different materials [55,56,57,58,59,60,61]. Especially, the estimate of the mechanical characteristics of concrete is very important as it requires a lot of time, effort, and cost to have the experimental results. To minimize these parameters, numerous AI algorithms such as random forest (RF), multi-linear perception regression (MLP), artificial neural network (ANN), neuro-fuzzy regression, AdaBoost, bagging, and boosting are normally used for the estimate of concrete properties. Shariati et al. [62] research was based on the anticipation of concrete strength containing waste material (FA and furnace slag). The result reveals that the ANN approach shows a satisfactory prediction level for the compressive strength (C-S) of concrete. Han et al. [63] employed the RF algorithm for the anticipation of high-performance concrete and described that RF could be successfully employed for the forecast of C-S of concretes. Chaabene et al. [64] represent a comprehensive review of the number of PML approaches used for the prediction of the strength properties of concrete. They reported that ML models are more precise, adaptable, and can be retrained by incorporating the updated dataset.

This study describes the combined effect of experimental and soft computing predictive approaches for the concrete strength containing FA. A detailed investigation of the material used and mix ratios for preparing the concrete were carried out for the desired strength. The novelty of this research is to investigate the precision level of predictive algorithms (MLP, DT, BR, RF) employed in the experimental and data retrieved from literature for the strength property of FA-based concrete. The comparative study on the precision of employed algorithms towards the prediction of C-S would be beneficial for the scientists and researchers in the field of engineering to adopt the appropriate technique for the estimate of concrete’s strength.

## 2. Materials and Methods

### 2.1. Materials

The materials utilized in this investigation were aggregates with a specific gravity of 2.79 and water absorption of 0.96% purchased from a local quarry, Ordinary Portland Cement Type I having a surface area of 380 m^2^/kg, and class-F fly ash obtained from a nearby thermal power plant was introduced in the experimental work. The water absorption for the selected fine aggregate was noted as 2.32%, with its specific gravity of 2.65 obtained from the local source. As per the ASTM standard C494 superplastizers type A was used in the concrete during experimental work. Table 1 summarizes the physical properties and chemical composition of cementitious materials. As can be observed, cement has the highest specific gravity, as opposed to FA. Moreover, the amount of SiO_2_, Fe_2_O_3_, and Al_2_O_3_ in FA is 77.9%, indicating that it is class-F FA. However, the fineness modulus FM of fine aggregate was noted as 2.65, while the result of fineness modulus for coarse aggregate and fine aggregate was calculated as 6.93, and 2.65, respectively.

### 2.2. Methods

In the laboratory, cylindrical specimens (100 mm diameter and 200 mm height) were made. Compaction was accomplished in two layers, with each layer receiving twenty blows, using a conventional 2.5 kg proctor hammer. This technique has been advocated over vibration and rodding. The number of random mixes was made with different mix ratios to obtain the maximum number of data points. Each batch was then subjected to curing for 7, 28, 56, and 90 days

### 2.3. Compressive Strength

The C-S of the FA-based concrete specimens was found using the ASTM C39/C 39M-99 standard [65]. The compressive axial load is applied to the specimens at a rate of 0.15 to 0.35 MPa/s until the failure. Concrete specimens were cured in water and then tested after 7, 28, 56, and 90 days. The maximum, minimum, and average C-S obtained from the experimental work in the laboratory were 60.90 MPa, 12.05 MPa, and 31.73 MPa, respectively.

### 2.4. Data Description 

**Figure 1:**
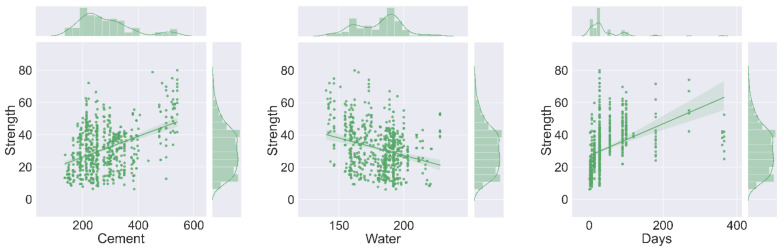

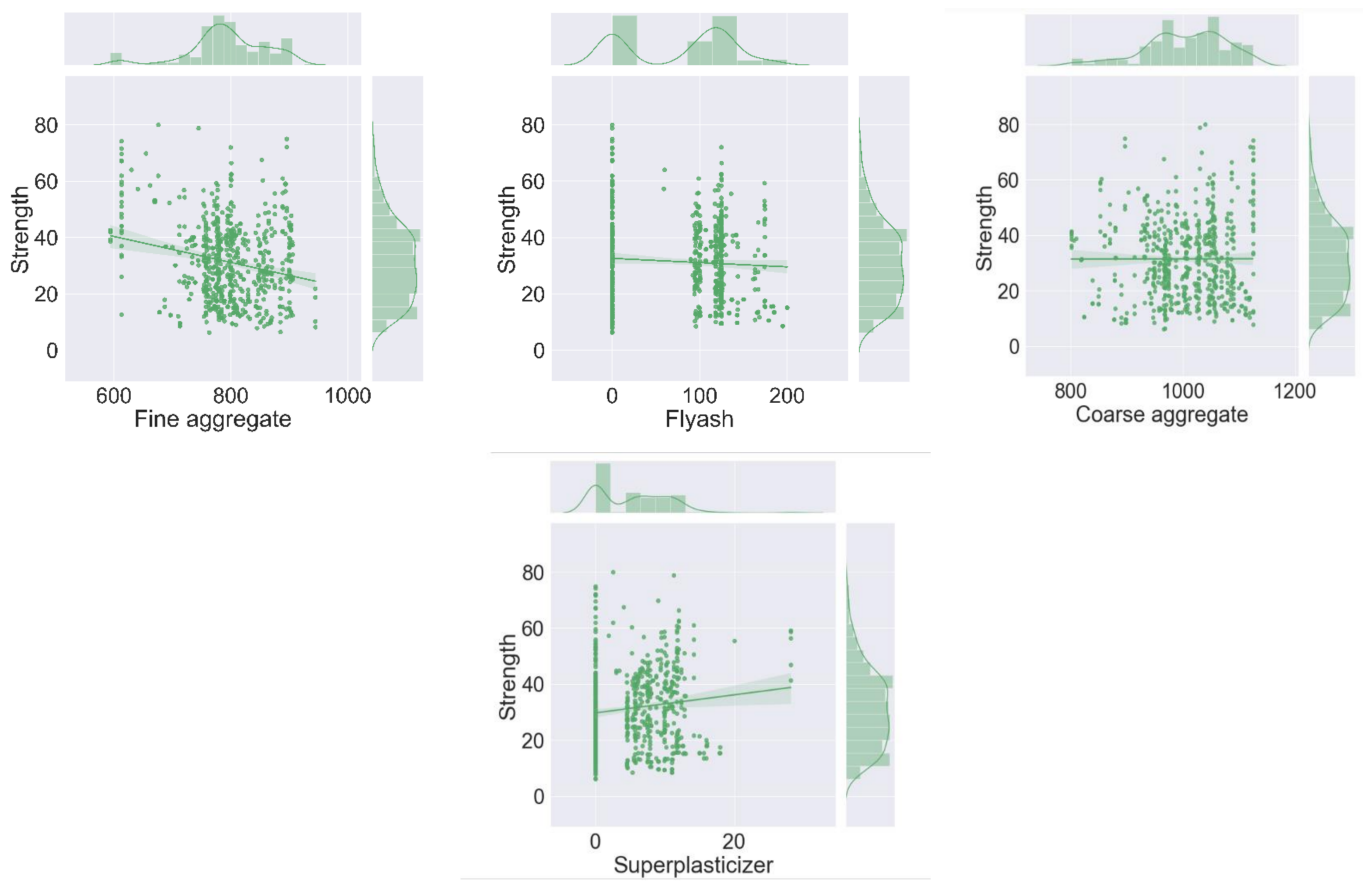
Reflection of histograms for inputs indicating the relative frequency distribution.

**Figure 2:**
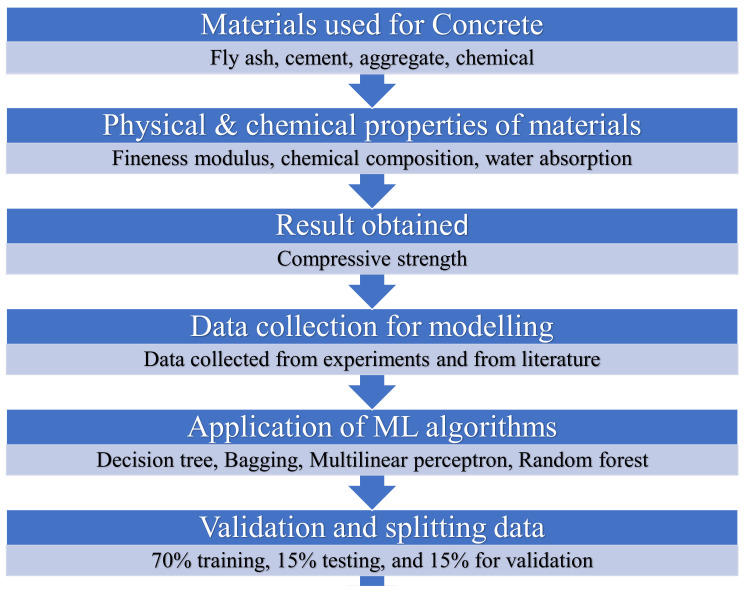
Flow chart of the research program indicating the step-by-step procedure.

The 62 data points (mixes) were prepared from the experimental work in the laboratory, while 569 data points were retrieved from the literature [66,67] to have a maximum number of data samples for modeling. To run the selected models, a total of 631 data points with seven input parameters such as FA, water (W), cement (C), superplasticizers (SP), age, coarse aggregate (C-A), and fine aggregate (FA), with one output C-S were arranged in the tabulated form. The required dataset was then incorporated into the anaconda navigator software, in which the selected models were run one by one with the help of python coding. The result was obtained in the form of a coefficient of determination (R^2^) value, which normally ranges from 0 to 1. The maximum R^2^ value signifies the superior precision level of the employed method in forecasting desired outcome. In addition, the explanatory statistical analysis of the input parameters obtained from experiments and literature used in the study for the prediction (C-S) purpose can be seen in Table 2. The histograms indicating the relative frequency distribution in the percentage of each variable of the total dataset were developed using Jupyter Notebook (6.0.3) of the anaconda software, as depicted in Figure 1 and the units for each variable in the figure is kg/m^3^, except age is days and strength in MPa. Moreover, the detailed schematic representation of this research is shown in Figure 2.

## 3. Predictive Machine Learning (PML) Algorithms

### 3.1. Decision Tree

**Figure 3:**
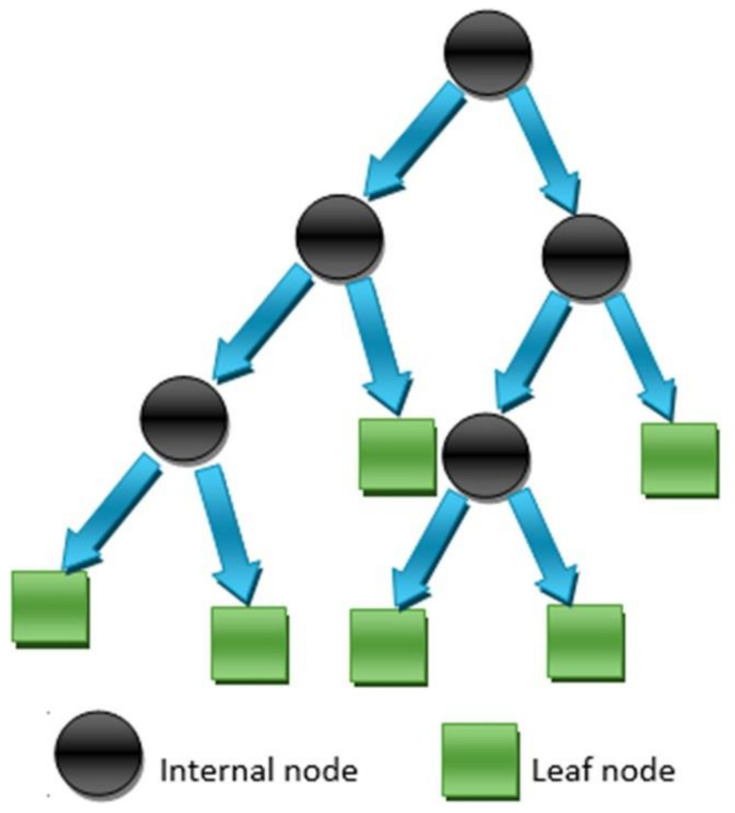
Execution process of the DT model [66].

DT algorithms are well-recognized PML approaches that have been used for a variety of tasks, most notably classification. DTs are used to partition datasets in a nonparametric manner. Alternative data extracting methods include regression models, which depict variables’ relations as cross-products. The DTs used in this research were chosen for their capacity to transform enormous, complex datasets into simple-to-understand yet knowledge-rich graphic presentations. More precisely, the resulting graphical tree image was deemed beneficial for rapidly elucidating the essential parameter value combinations that result in unacceptable product loss, which could then be turned into a set of rules. A DT employs a tree-like graph to describe a flowchart-like structure, with the “root” as the starting point. Each internal node of the tree corresponds to a test on a particular attribute or subset of attributes. Each branch from the node reflects the result of the test, while the final node represents a class label via a “leaf”. A simple DT can be constructed manually. However, designing an algorithm that learns the tree from data is straightforward. As with other types of PML, supervised learning uses labeled samples to construct a classifier by computing the sequence of branch options. The flow chart of the DT model indicating the execution process for predicting the required outcome is shown in the Figure 3.

### 3.2. MLP Algorithm

**Figure 4:**
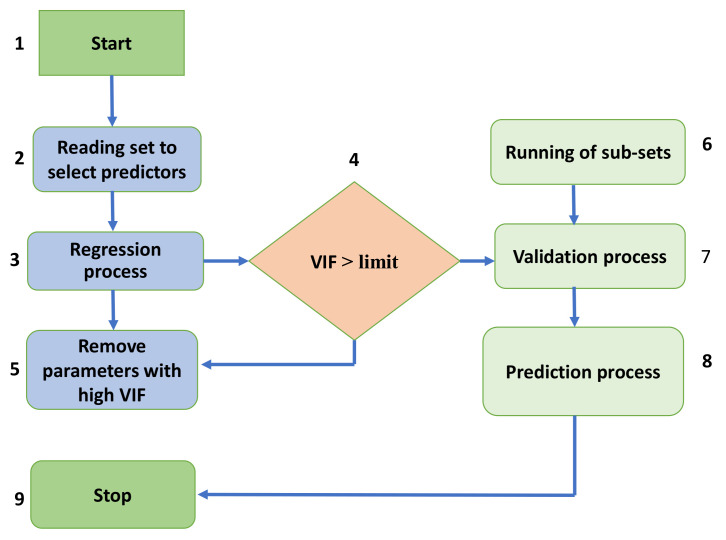
Flow chart of the MLP model showing the complete execution process.

An MLP is a form of feedforward ANN that generates outputs based on a collection of inputs. Amongst the output and input layers, many layers of input nodes are linked through a targeted graph. Backpropagation is used to train the network in MLP. A MLP is a type of network (neural) that links many laps in a targeted graph, with signals traveling one way across the nodes. Except for the input nodes, each node has a nonlinear activation function, which is unique to it. MLPs are a type of supervised learning that makes use of backpropagation. Due to the number of laps of neurons in MLP, it is usually called a deep learning approach. MLP is commonly used in supervised learning applications and imputation pure science and parallel dispersed processing studies. Applications have machine translation, image perception, and speech realization. Initially, the algorithm selects predictors to employ during the regression phase to identify the variance inflation component (VIF). The VIF then evaluates the variance increase of an estimated regression coefficient due to collinearity. Finally, the algorithm eliminates variables with high VIFs in order to get the optimal forecasting solution as shown in the Figure 4.

### 3.3. Bagging Algorithm

**Figure 5:**
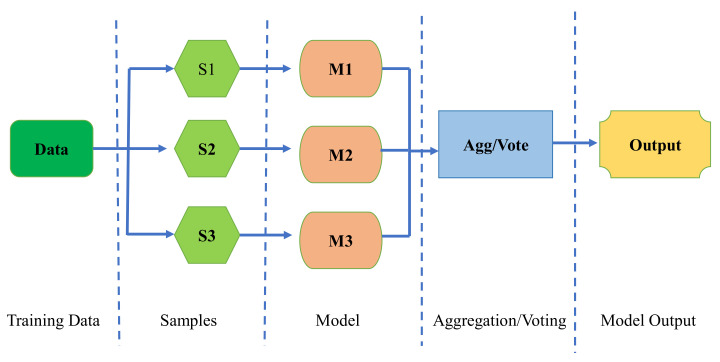
Flow chart of the bagging algorithm indicating the execution process.

BR, also known as bootstrap aggregation, is a technique for merging many editions of a predicted model. Every model is individually skilled and then averaged. The fundamental purpose of BR is to achieve a smaller deviation than any single model. Bootstrapping is the process of generating bootstrapped samples from a given dataset. The samples are generated by randomly picking and replacing data points. The resampled data have qualities that are unique from the original data in their entirety. It illustrates the data distribution and also tends to reserve divergence among bootstrapped samples, i.e., the data dispersal must remain together while maintaining distinction across bootstrapped samples. This helps to construct strong models. Furthermore, bootstrapping supports preventing the overfitting problem. When several training datasets are used to build the model, it becomes resistant to error creation and hence runs in a better manner with the test data, minimizing variation by creating a strong footing in the test set. Testing the model with numerous permutations guarantees that it is not partisan for an incorrect result. The flow chart of the bagging model can be seen in the Figure 5.

### 3.4. Random Forest

**Figure 6:**
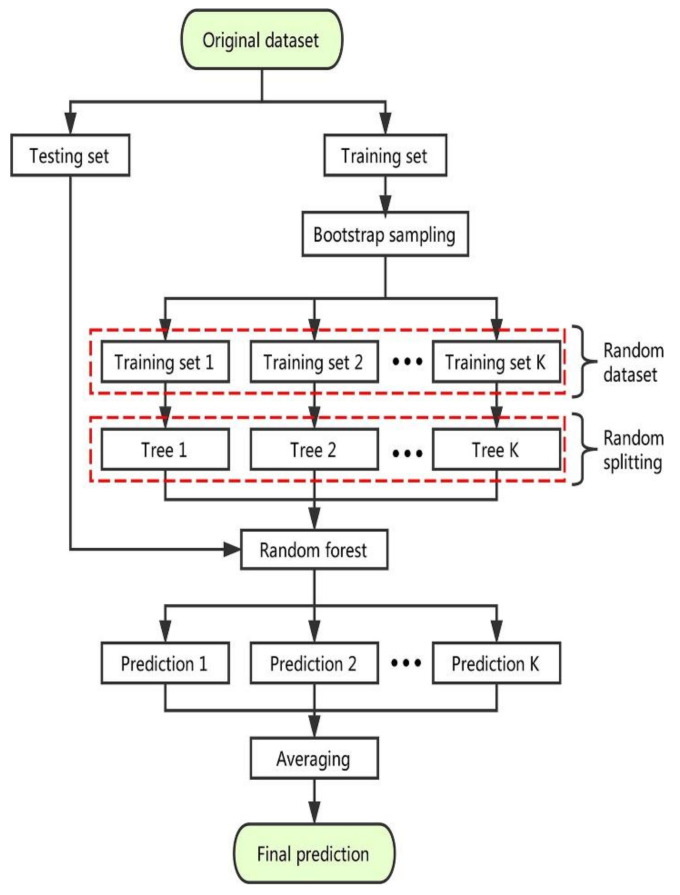
Predictive process of the RF model [63].

An RF is a special kind of PML method that is utilized to deal with classification and relapse issues. It constructs the use of ensemble learning, a practice for settling complex problems through the application of various classifiers. An RF algorithm is made up of a huge number of decision trees. The RF approach creates a ‘forest’ that is trained using either backward regression or bootstrap aggregation. BR is an ensemble meta-algorithm that is used to improve the accuracy of PML systems. The RF technique creates the result based on the predictions of the DTs. Forecasting is accomplished by summing or scaling the output of distinct trees. Expanding the number of trees enhances the accuracy of the result. An RF algorithm solves the disadvantages of a deep learning system. It reduces overfitting and increases the accuracy of datasets. It makes predictions without needing the user to configure multiple packages (such as sci-kit-learn). A DT is composed of three components: decision nodes, leaf nodes, and root nodes. A DT technique partitions a training set into branches that subsequently split into additional branches. This method is continued till reaching a leaf node. It is not feasible to further segregate the leaf node. The nodes of the DT show the attributes that are used to anticipate the result. The decision nodes link the leaves together. The execution process of the RF model is depicted in the Figure 6.

### 3.5. K-Fold Cross-Validation (C-V) Method

C-V is a statistical approach that is used to assess the prediction power of PML models. It is commonly applied in PML to match and select models for specific projecting modeling issues since it is simpler to understand and use and gives skill estimates that are typically less biased than those given by other approaches. C-V is a strategy for assessing PML models on a short sample of data. The method accepts a single parameter, k, which indicates how many groups a given data sample should be split into. As a result, the procedure is usually abbreviated as k-fold C-V. When an exact value for k is supplied, it may be used in place of k in the model’s reference; for example, k = 10 becomes 10-fold C-V.

C-V is mostly utilized in applied PML to determine the skill of a PML model on formerly unknown data. That is, to assess the model’s overall operation when employed to produce forecasts on data that were not used during the model’s training. It is a popular method because it is simple to understand and offers a more accurate evaluation of model competency than other strategies, such as a simple train/test split. The general procedure is as follows: randomize the dataset, divide it into k distinct groups, treat one group as a reserve or test data collection, use the remaining groups as a source of training data on the training set, fit a model and evaluate it on the test set, keep the evaluation score and discard the model, and summarize the model’s ability by examining a sample of model evaluation scores. Notably, each observation in the data sample is assigned to a unique group and remains assigned to that group throughout the process. This means that each sample is used just once in the hold outset and then used k times to train the model.

## 4. Result and Discussions

### 4.1. Decision Tree Model Outcome

**Figure 7:**
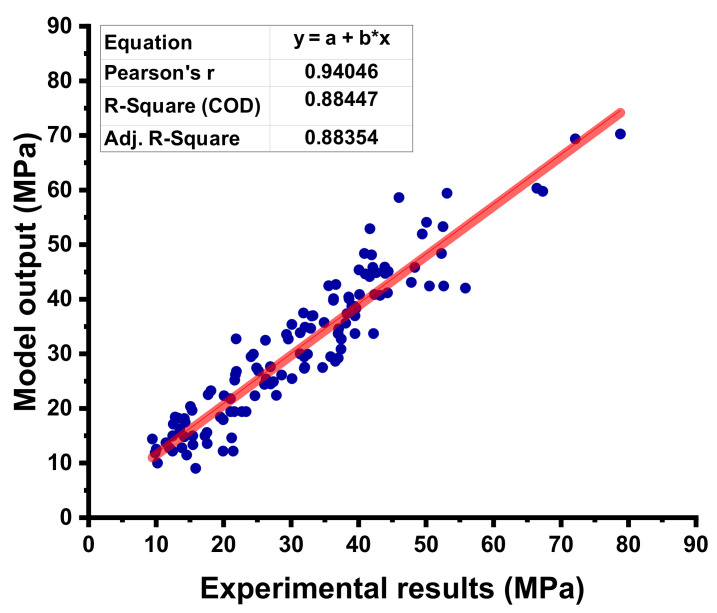
Correlation between the experimental C-S and projected C-S for the DT model.

**Figure 8:**
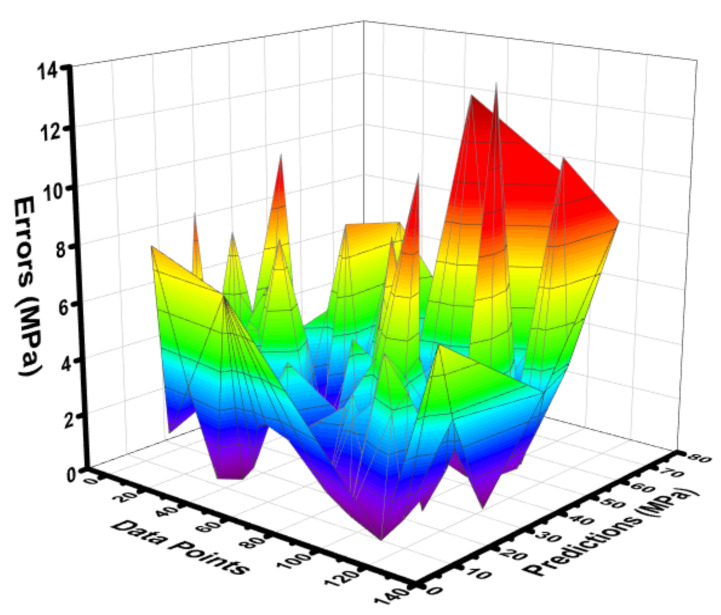
Difference between the experimental C-S and predicted C-S of the DT model.

The correlation amongst the experimental results and the findings found from the DT model (predicted) shows appreciable relation and gives the R^2^ value equal to 0.88, as shown the Figure 7. However, Figure 8 depicts the spreading of errors from the predicted and experimental C-S results. This distribution ranges from 0 and gives the maximum value equal to 13.8 MPa, while the average result of this distribution was 3.09 MPa. In addition, 23.62% of the data were lying among 0 and 1 MPa, and 58.26% of the data were lying among 1 MPa and 5 MPa. However, only 18.11% of the error values were lying above 5 MPa.

### 4.2. MLP Model Outcome

**Figure 9:**
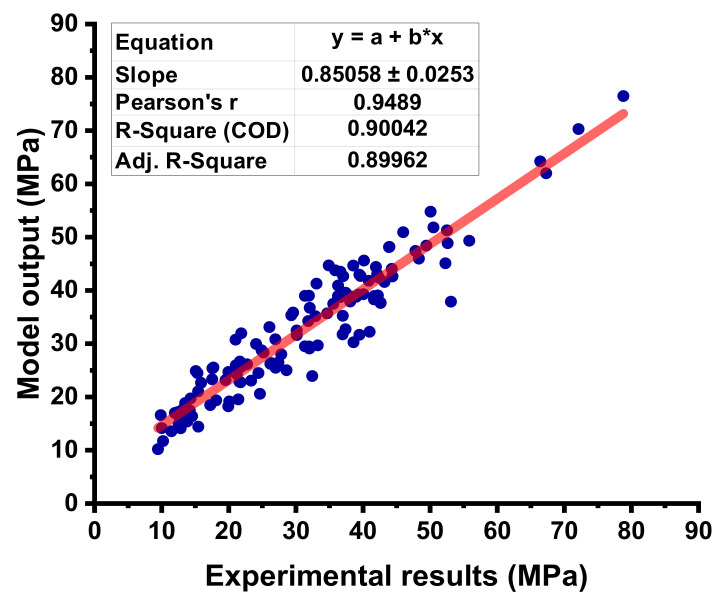
Correlation between the experimental C-S and the estimated C-S for the MLP model.

**Figure 10:**
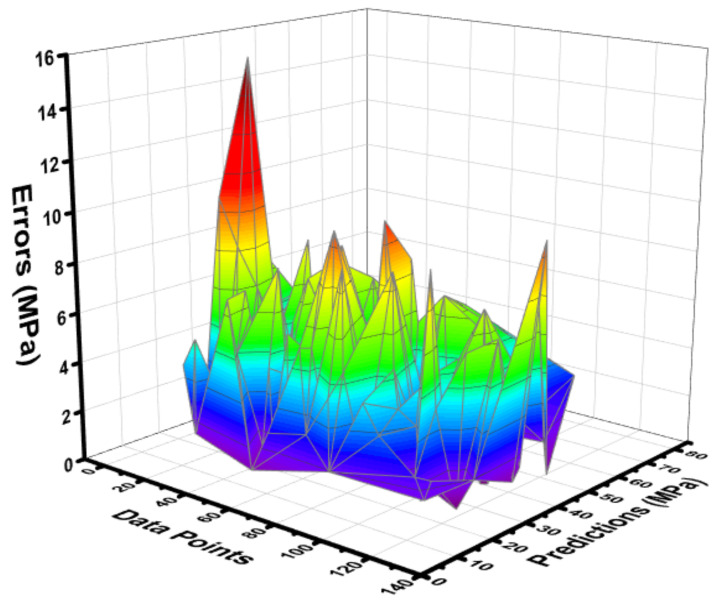
Difference between the experimental C-S and predicted C-S of the MLP model.

The statistical result obtained from the MLP model between the experimental and predicted can be seen in Figure 9. The R^2^ value equals 0.90 for the MLP model, showing a better predictive precision for C-S of concrete as opposed to the DT model. The difference (errors) between the experimental and forecasted C-S results for FA-based concrete are shown in Figure 10. This difference gives the maximum value equal to 15.22 MPa, while the minimum value was reported as 0.009 MPa, while this distribution shows the average value equals 3.74 MPa. Moreover, it was reported that 14.17% of data were lying up to 1 MPa, and 56.69% of data were lying among 1 MPa and 5 MPa. However, 29.13% of the data were lying above 5 MPa.

### 4.3. BR Model Outcome

**Figure 11:**
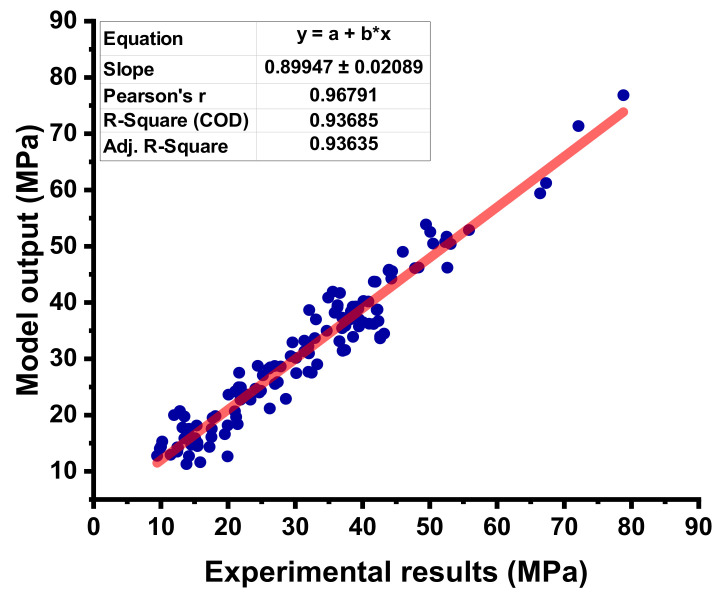
Correlation between the experimental C-S and projected C-S for the BR model.

**Figure 12:**
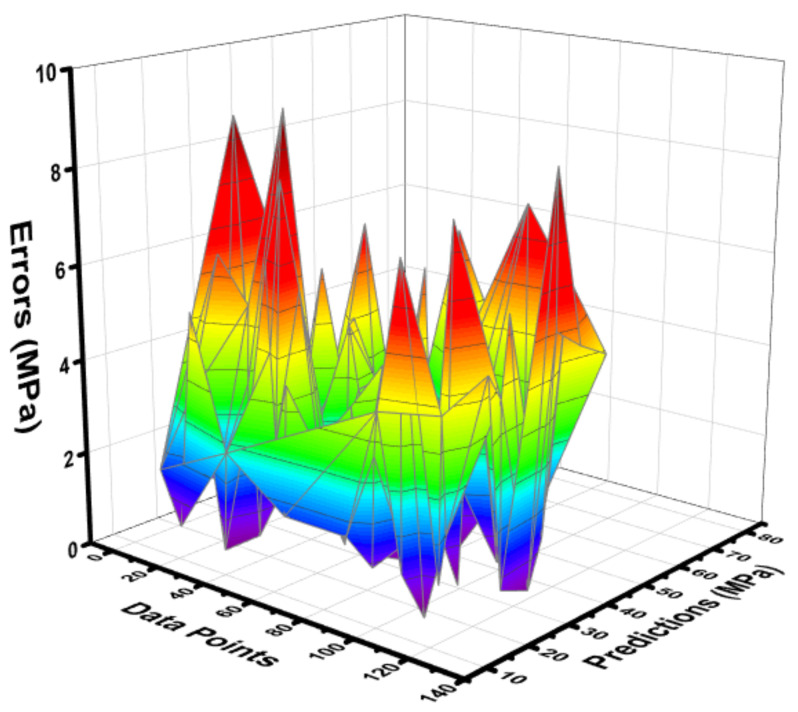
Difference between the experimental C-S and predicted C-S of the BR model.

The relationship between the experimental results of the C-S and the anticipated outputs of the concrete containing FA are shown in Figure 11. The results of the difference (errors) among the forecasted and experimental can be seen in Figure 12. The results of these differences give the highest, lowest, and average values of 9.01 MPa, 0.004 MPa, and 2.77 MPa, respectively. Moreover, 23.62% of the data were lying up to 1 MPa, 59.05% of data were found among 1 MPa, and 5 MPa, while only 17.32% of the data were lying above 5 MPa.

### 4.4. RF Model Output

**Figure 13:**
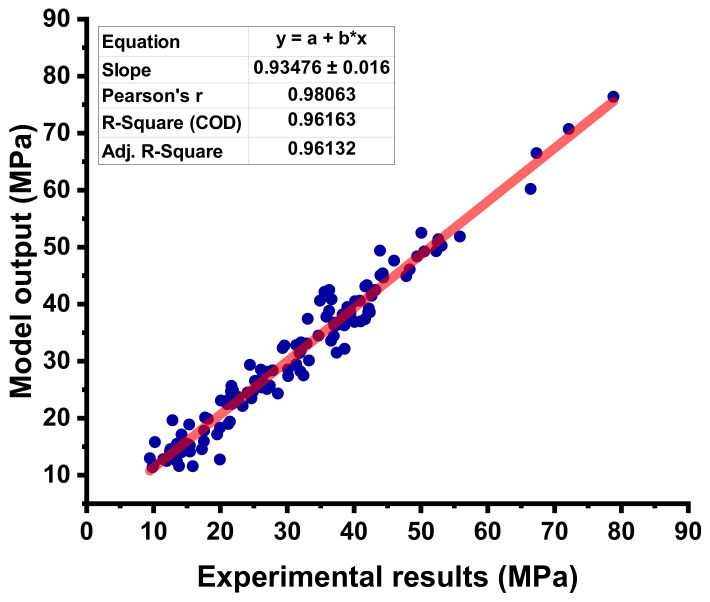
Correlation between the experimental C-S and projected C-S for the RF model.

**Figure 14:**
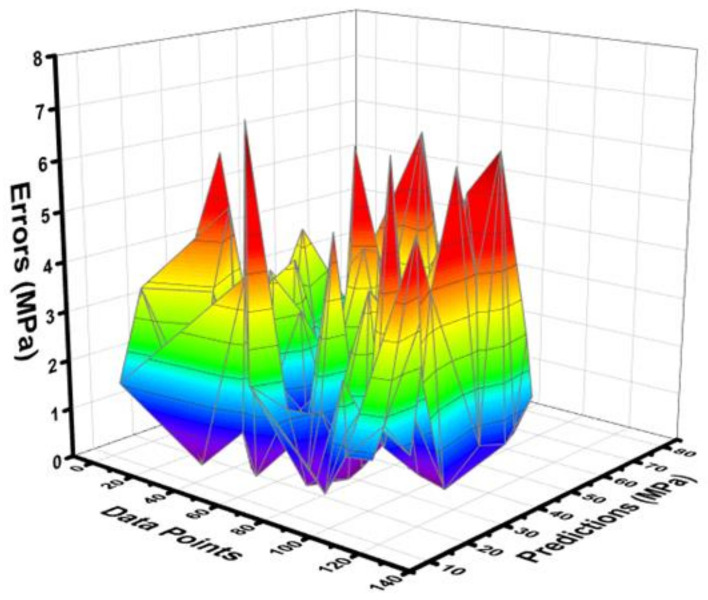
Difference between the experimental C-S and predicted C-S of the RF model.

The statistical output for the RF model between the experimental C-S and predictive C-S of concrete containing FA is depicted in Figure 13. The RF model shows a much better predictive result when compared to other employed ML algorithms, as illustrated by the high R^2^ value that equals 0.96. The errors distribution between the experimental C-S and forecasted C-S of concrete is shown in Figure 14. The RF model’s error distribution gives the highest, lowest, and average values equal to 7.183 MPa, 0.056 MPa, and 2.170 MPa, respectively. Moreover, it was observed that 24.40% of the data were lying up to 1 Mpa, 67.71% of the data were lies among 1 MPa, and 5 MPa, while only 7.87% of the data were lying above 5 MPa.

### 4.5. K-Fold Outcome

C-V is a statistical approach that is used to analyze or approximate the factual performance of PML models in real-world situations. It is crucial to understand the effectiveness of the models that have been chosen. In order to accomplish this, a validation technique must be used to determine the level of correctness of the model’s data. The k-fold validation test necessitates the randomization of the dataset as well as the division of the dataset into k-groups. According to the research detailed here, the data from experimental samples are separated into ten equal groups. It makes use of nine out of ten subsets, with the exception of one subset that is used for model validation purposes. The same approach used in this process is then replicated ten times in order to get the average precision of the ten replications carried out. It has been extensively established that the k-fold C-V approach accurately depicts the decision and correctness of the PML models, and this has been thoroughly confirmed.

**Figure 15:**
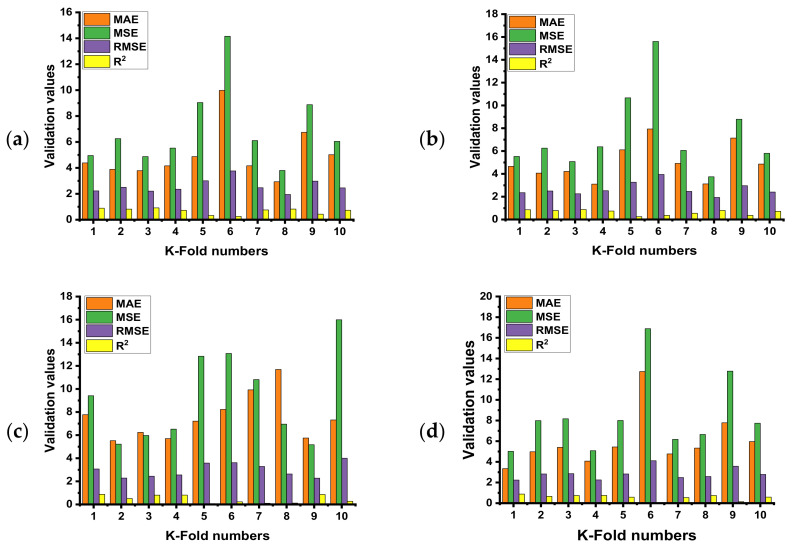
Statistical indicators of k-fold CV for the employed models; (**a**) RF model, (**b**) BR model, (**c**) MLP model, and (**d**) DT model.

The use of k-fold C-V might be employed to determine whether or not there is a bias or a variance reduction for the test set. As shown in Figure 15a–d, the outcomes of C-V are assessed using the R^2^, the mean absolute error (MAE), the mean square error (MSE), and the root mean square error (RMSE). The RF model indicates the lower result of the proposed errors and high result of the R^2^ as opposed to the other three employed models (BR, MLP, DT). RF shows the average value of R^2^ equals 0.46, while the maximum and minimum values were equal to 0.88 and 0.07, respectively. The BR model’s average R^2^ value was noted as 0.63, and the highest and lowest value was reported as 0.87 and 0.25, respectively. Likewise, the average, least, and high value of R^2^ for the MLP model was noted as 0.47, 0.07, and 0.88, respectively. However, the same result of the R^2^ value for the DT model was reported as 0.57, 0.01, and 0.88, respectively.

## 5. Sensitivity Analysis (SA)

**Figure 16:**
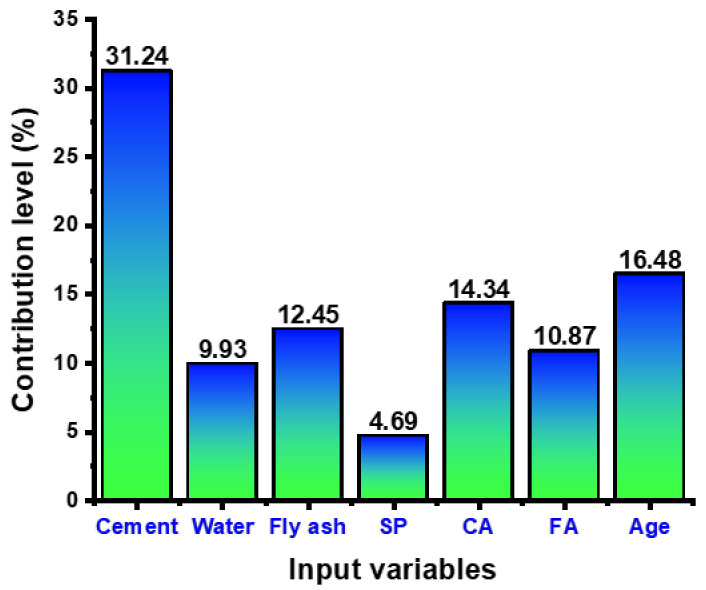
Parameter’s effect on the strength property of FA-based concrete.

This analysis helps to find out the contribution level of each input factor employed for modeling to predict the C-S of FA-based concrete. It is also important to test the effect of each variable for the required outcome. SA reveals that the highest contribution towards the prediction of C-S was reported by cement and shows the 31.24 percent contribution, while the other variables contributed the least. The minimum contribution was reported by the superplasticizers, which contributed only 4.69 percent towards the anticipation of C-S of concrete, as shown in Figure 16.

## 6. Discussion

**Figure 17:**
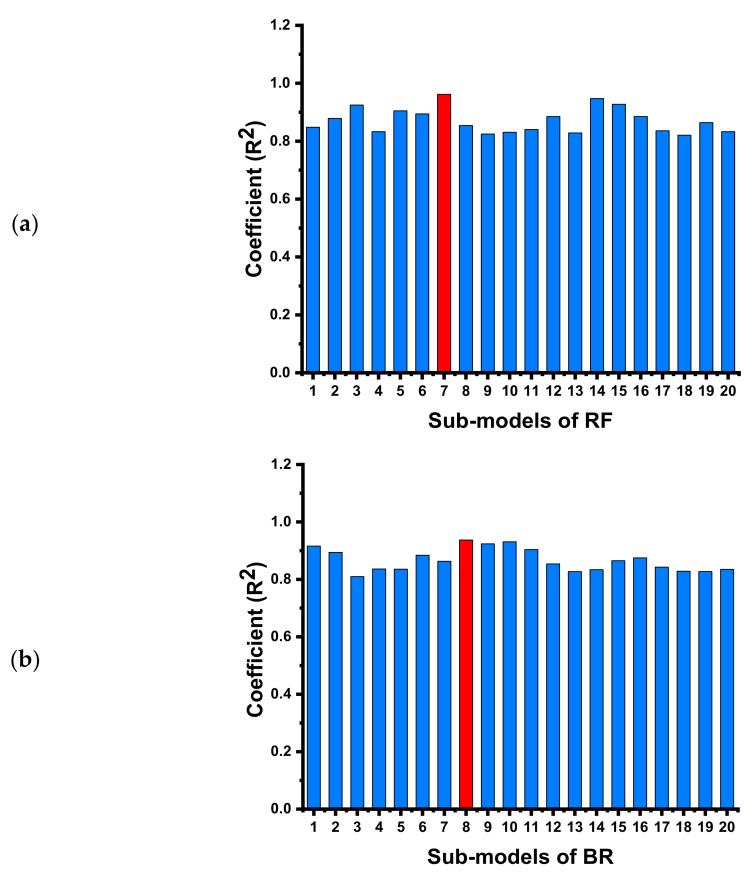
Coefficient of determination result of the 20 sub-models for; (**a**) RF, (**b**) BR models.

This research described the comparative investigation of experimental results obtained in the laboratory and forecasted results acquired from the various modeling techniques for the C-S of concrete containing FA. It is the worth known fact that obtaining the strength of concrete must take a number of days (time), which is a time-consuming effort for researchers. To minimize time, effort on experiments, and cost, the application of such soft computing methods which can predict the desired strength initially are of great interest. The ML algorithms employed in this study also showed satisfactory outcomes when the experimental C-S result of the various mixes was compared with the forecasted C-S result. The comparison of four different types of ML approaches gives the anticipated result with a certain precision level based on the execution process of each approach. The RF ML technique gives the effective, precise result for C-S of FA-based concrete when compared to other employed ML algorithms (DT, MLP, and BR). The precision level of these models is normally evaluated from the R^2^ value, which normally ranges from 0 to 1; the higher R^2^ value of the model indicates a better precise result in terms of predictions. The high accuracy of the RF and BR is due to the execution process for the data and splitting of the model into the sub-models. The detailed information on the sub-models of RF and BR can be seen in Figure 17a,b, respectively. An RF is composed of a huge number of independent DTs that involve collaboration. Each tree in the RF produces a forecast for a class, and the class with the most choices becomes the model’s prediction. The high accuracy of the RF model over the others has also been reported in the literature [68]. The applied statistical checks also give confirmation of high accuracy for the RF model. The lesser value of MAE, MSE, and RMSE shows that the R^2^ value for the said model will be higher and vice versa.

## 7. Conclusions

This research reported the comparative study of experimental C-S and the results from the various modeling approaches for concrete containing fly ash (FA). The 61 mixes were prepared in the laboratory with the random mix ratios to have the number of data points for further investigation in the modeling techniques. A similar database was also collected from the literature to make the database appreciable for modeling. The following conclusion can be drawn from the study.

- The RF model was more effective in predicting the C-S of concrete having FA as opposed to DT, MLP, and BR.
- RF gives the R^2^ value equal to 0.96, which is the highest of the DT (0.88), MLP (0.90), and BR (0.93), indicating the highest precision level for forecasting the C-S of concrete.
- Statistical checks and the CV approach also validate the superior exactness level of the RF model as opposed to other employed models.
- The RF also gives a lesser result for the evaluated errors MAE (2.17 MPa), MSE (7.45 MPa), and RMSE (2.73 MPa) when compared with the error value of the DT, MLP, and BR. This lesser value of the error also confirms the high precision of the RF model.

Further studies can also be conducted using other supervised ML algorithms such as boosting regressor, Adaptive neuro-fuzzy inference system, and XGBoost technique to investigate their predictive performance. Furthermore, the experimental approach can also be enhanced to obtain the maximum number of data points to avoid overfitting the data. It is also recommended that the strain model can also be included in the study along with the use of supervised machine learning algorithms to strengthen the overall quality of research work. To compare the results with a database with restricted input parameters, the number of input variables might be expanded. The dimensions of the tested specimens, temperature, and humidity effects can also be considered to investigate the difference in the required outcome.

## Figures and Tables

**Table 1:** Physical properties and chemical composition of the cement and fly ash.

	Property/Composition	Cement	Fly Ash
Physical properties	Initial setting time (minutes)	34	-
	Final setting time (minutes)	161	-
	Standard consistency (%)	31.9	-
	Specific gravity	3.2	2.482
	Soundness (mm)	1	-
	Blaine fineness (m^2^/kg)	2950	4300
Chemical composition	Silica as SiO_2_ (%)	21.77	48.5
	Alumina as Al_2_O_3_ (%)	5.5	20.01
	Magnesium as MgO (%)	1.24	2.4
	Calcium as CaO (%)	63.3	16.45
	Iron as Fe_2_O_3_ (%)	4.6	8.5
	Sulphur as SO_3_ (%)	1.91	1.72
	Loss of ignition (%)	1.68	2.42

**Table 2:** Explanation of the statistical analysis for the input parameters.

Parameters	Cement (kg/m^3^)	Fly Ash (kg/m^3^)	Water (kg/m^3^)	SP (kg/m^3^)	C-A (kg/m^3^)	FA (kg/m^3^)	Age (days)
Mean values	282.13	77.29	180.95	5.45	1003.76	794.19	44.50
Standard deviation	94.88	61.91	17.97	5.28	72.84	68.18	58.66
Median of input	252.00	100.40	185.70	5.70	1006.40	794.90	28.00
Mode of input	213.50	0.00	192.00	0.00	968.00	613.00	28.00
Standard error	3.78	2.46	0.72	0.21	2.90	2.71	2.34
Range	405.30	200.10	88.00	28.20	324.00	351.00	364.00
Minimum values	134.70	0.00	140.00	0.00	801.00	594.00	1.00
Maximum values	540.00	200.10	228.00	28.20	1125.00	945.00	365.00

## Data Availability

The data used in this research has been properly cited and reported in the main text.
